# Multiple-Micronutrient Fortified Non-Dairy Beverage Interventions Reduce the Risk of Anemia and Iron Deficiency in School-Aged Children in Low-Middle Income Countries: A Systematic Review and Meta-Analysis ^(i–iv)^

**DOI:** 10.3390/nu7053847

**Published:** 2015-05-21

**Authors:** Grant J. Aaron, Daphna K. Dror, Zhenyu Yang

**Affiliations:** 1 Global Alliance for Improved Nutrition (GAIN), Rue de Vermont 37-39, Geneva CH-1202, Switzerland; 2 United States Department of Agriculture (USDA), Western Human Nutrition Research Center (WHNRC), 430 W. Health Sciences Dr., Davis, CA 95616, USA; E-Mail: daphnadror@gmail.com; 3 Key Laboratory of Trace Element Nutrition, Ministry of Health of China, National Institute for Nutrition and Health, Chinese Center for Disease Control (CCDC), No. 29 Nanwei Road, Xicheng District, Beijing 100050, China; E-Mail: yang.zhenyuid@gmail.com

**Keywords:** fortification, multiple-micronutrients, beverages, children, low-income

## Abstract

Multiple-micronutrient (MMN) fortification of beverages may be an effective option to deliver micronutrients to vulnerable populations. The aim of the present systematic review and meta-analysis is to evaluate the nutritional impacts of MMN fortified beverages in the context of low-middle income countries. A systematic search of published literature yielded 1022 citations, of which 10 randomized controlled trials (nine in school-aged children and one in pregnant women) met inclusion criteria. Results of school-aged children were included in the meta-analysis. Compared to iso-caloric controls, children who received MMN fortified beverages for 8 weeks to 6 months showed significant improvements in hemoglobin (+2.76 g/L, 95% CI [1.19, 4.33], *p* = 0.004; 8 studies) and serum ferritin (+15.42 pmol/L, [5.73, 25.12], *p* = 0.007; 8 studies); and reduced risk of anemia (RR 0.58 [0.29, 0.88], *p* = 0.005; 6 studies), iron deficiency (RR 0.34 [0.21, 0.55], *p* = 0.002; 7 studies), and iron deficiency anemia (RR 0.17 [0.06, 0.53], *p* = 0.02; 3 studies). MMN fortified beverage interventions could have major programmatic implications for reducing the burden of anemia and iron deficiency in school-aged children in low-middle income countries. Additional research is needed to investigate effects on other biochemical outcomes and population subgroups.

## 1. Introduction

In resource poor settings, vulnerable population groups including children and women of reproductive age are prone to multiple micronutrient (MMN) deficiencies primarily due to relatively high requirements, poor dietary quality [1] and/or increased susceptibility to infection [2,3]. For example, globally iron deficiency anemia affects 18% and 19% of children under-5 years and pregnant women respectively, while 35% of children under-5 years and 15% of pregnant women suffer from vitamin A deficiency. Iodine and zinc deficiencies are estimated to affect 29% and 17% of the world’s population [4]. During pregnancy and early childhood, MMN deficiencies increase maternal, infant, and child morbidity and mortality and prevent surviving offspring from attaining full developmental capacity [4]. Interventions that deliver MMN to these populations are therefore warranted, both to enhance nutritional outcomes compared to single micronutrient interventions [1], and to increase cost-effectiveness of delivery [5]. 

The two most widely used strategies for delivering additional micronutrients are: (1) supplementation—defined as the provision of micronutrients in capsule, tablet, or syrup form; and (2) fortification—for the purposes of the current paper defined as micronutrients added to processed food vehicles, or at point-of-use (home fortification) [6,7]. The appropriateness, extent of use, and programmatic success of these approaches vary considerably by country and context, depending on the population subgroups targeted, the nature and severity of deficiencies, as well as the financial and technical resources to develop and sustain delivery [8]. Supplementation and fortification are generally considered complementary strategies, with the former most suitable in contexts where certain population sub-groups may not be reached, or when nutrient requirements may not be met by other intervention strategies. Fortification has several advantages in terms of consumer acceptance, relative ease of fortifying foods, reach and coverage, cost-effectiveness, and sustainability [6]. Numerous international organizations, governments, consensus meetings, and more recently the Scaling Up Nutrition (SUN) movement, have endorsed fortification as one of the key strategies to reduce MMN deficiencies in high-burden countries [9]. Most programmatic experience in this context to date has focused on large-scale fortification of staple foods targeted to the general population, and more recently to home (or point-of-use) fortification products targeted to infants and young children. MMN fortification of beverages, though not widely implemented, may be an effective option to cover gaps in other existing nutrition strategies. Some advantages of beverages as vehicles for fortification include high consumer acceptance, achieved via potential fortification of familiar beverages, and flexible delivery as a ready-to-consume (RTC) beverage products or as powder which can be reconstituted [10,11,12]. 

The aim of the present systematic review and meta-analysis is to review the nutritional impacts of non-dairy fortified beverages in the context of low-middle income countries. The primary focus of the review is on school aged children due to the paucity of studies in other population groups. Dairy beverages were excluded to limit potential confounding effects of other nutritional factors present in milk products in the absence of non-dairy MMN fortified beverage control groups. 

## 2. Methods

### 2.1. Search Strategy

The systematic review was conducted in line with the guidelines for Preferred Reporting Items for Systematic Reviews and Meta-Analyses (PRISMA). A systematic search of published literature from 1 January 1966 through 28 February 2013 was undertaken, and subsequently updated on 15 February 2015, using the US national Library of medicine’s MEDLINE/PubMed bibliographic search engine. Multiple PubMed searches were conducted using various combinations of Medical Subject Heading (MeSH) and Title/Abstract (TIAB) keywords (Table 1). A filter was used to select only studies conducted on humans; no filters were set for language restrictions. All co-authors independently screened unique article titles and abstracts using a standardized form to identify a short list of studies for further review. When opinions differed, discrepancies were resolved by co-author consensus or by two-thirds majority if consensus was not achieved. The same process as above was carried out to identify the subset of articles to include for qualitative review. For those articles included in the qualitative review, the following data were extracted: setting, study population, study design, duration, intervention formulation, placebo formulation, frequency of beverage administration, and outcomes assessed. For biochemical and hematological outcomes, all data except hemoglobin were converted to international system (SI) units. 

**Table 1:** PubMed search strategy.

Step	Search Strategy 1 ^1^	Search Strategy 2 ^1^
1	“beverages” [MeSH] ^2^ NOT “milk” [MeSH]	Beverage * ^3^ [TIAB] ^4^ NOT milk * [TIAB]
2	“food, fortified” [MeSH]	Fortif * [TIAB]
3	1 AND 2	1 AND 2
4	“beverages” [MeSH] NOT “milk” [MeSH]	Beverage * [TIAB] NOT milk * [TIAB]
5	“micronutrients” [MeSH]	Micronutrient * [TIAB] OR vitamin * [TIAB] or mineral * [TIAB] OR iron [TIAB] OR vitamin A [TIAB] OR zinc [TIAB]
6	4 AND 5	4 AND 5
7	3 OR 6	3 OR 6

^1^ All searches were performed in PubMed using a “humans only filter”; ^2^ MeSH: Medical Subject Heading; ^3^ Asterisk (*) is used to search for all terms beginning with the same word root; ^4^ TIAB: Title/Abstract.

In order to identify any additional articles not found by the PubMed searches, the reference sections for full articles reviewed were searched, and subject matter experts were consulted. An external scientific advisory board consisting of two subject matter experts was consulted to ensure that no related articles were missed in our analyses.

### 2.2. Study Eligibility Criteria

Studies considered for selection assessed the effects of non-dairy MMN fortified beverages (RTC or powders for reconstitution) on micronutrient status, anthropometry, morbidity, and/or functional outcomes. Only single or double blind randomized controlled trials (RCT) with treatment and placebo groups conducted in the context of low-middle income countries, on apparently healthy children and women of reproductive age were considered. No restrictions were made for the number of study arms, micronutrient formulations, or study duration. 

### 2.3. Statistical Analyses

For continuous outcomes, pooled SDs of the change were calculated if the change from baseline to end line and their SDs were reported. Final sample sizes were used to calculate the pooled SD if the number of participants decreased during the course of the study. If the change was not reported, the difference in final value between the intervention and control group was calculated and the SD of the final values from each group was used to calculate pooled SD. If the SD was not reported, 95%-CI of mean values were converted to SD; or medians with 25th and 75th percentiles were converted to mean and SD. For binary data, risk ratios and 95%-CI were calculated. Differences were considered significant at α= 0.05.

For trials with more than two groups, the comparison group most similar to MMN-fortified beverage *vs.* iso-caloric control was selected for the analysis [13,14]. One trial involved a 12-month intervention with results reported as change from 0–6 and 6–12 months [15]. For comparability with other designs only results from the 0–6 month period were included in the meta-analysis.

### 2.4. Assessment of Heterogeneity, Sensitivity, and Risk of Bias

A random-effects model was used to account for heterogeneity across studies, with I^2^ statistic calculated for each meta-analysis. Heterogeneity cutoffs were classified as: low < 25%; moderate 25%–75%; and high > 75% [16]. Meta-regression analyses were performed to evaluate relationships between predetermined independent factors: micronutrient dose, intervention duration, baseline values, deworming, and the most commonly reported outcomes (hemoglobin, serum ferritin or weight). Study bias was assessed by publication bias, the method of randomization, type of blinding (single or double), the percentage of loss to follow-up (low *vs.* high) and subgroup analyses.

## 3. Results

### 3.1. Search Results and Study Characteristics

The PubMed literature searches yielded 1158 citations updated on 15 February 2015. After removing duplicates (*n* = 136), titles and abstracts of 1022 citations were screened. Of these citations, 996 studies were excluded for various reasons (Supplemental Figure S1). Full texts were screened for 26 studies, of which 9 studies were included in the initial qualitative review. The reference sections for full texts yielded no additional studies. One co-author found a relevant study that was not yet indexed by PubMed after the initial qualitative review [11], which was eligible and subsequently included in the systematic review. 

The final set of 10 studies included a total of 4645 participants who had both baseline and end line measurements (Table 2). Studies were conducted in Bangladesh, Botswana, India, Nigeria, the Philippines, South Africa, and Tanzania. All studies were double-blind RCT; 9 trials were conducted in school children [10,11,13,14,15,17,18,19,20] and one trial was conducted in pregnant women [21]. The mean age range of children participating in the included studies was 5–18 years, and the mean age of women in the adult trial was 25 years. Eight of the trials had two study groups (MMN fortified beverage and iso-caloric control beverage), one trial also included a non-intervention control group, and one trial had four study groups including a MMN-fortified non-caloric beverage and an unfortified non-caloric control. End line sample sizes for the selected studies ranged from 89–989 (median 331; IQR 247–714), and study durations ranged from 2–12 months (median 4; IQR 2–6). Eight of the trials tested beverage powders, and two trials tested RTC beverages; various MMN formulations were tested (Supplemental Table S1). The selected trials evaluated the following outcomes: hemoglobin (*n* = 9); ferritin (*n* = 9); retinol (*n* = 6); zinc (*n* = 4); vitamin B12 (*n* = 3); folate (*n* = 3); vitamin C (*n* = 2); riboflavin (*n* = 2); vitamin B6 (*n* = 1); thiamin (*n* = 1); niacin (*n* = 1); iodine (*n* = 1); anthropometry (*n* = 8); cognition (*n* = 3); and physical performance (*n* = 2).

### 3.2. Methodological Quality

Methodological quality was considered moderate for 7 of 10 studies following downgrades for inadequate randomization or allocation concealment, high loss to follow up, or poor generalizability. Quality of the remaining individual trials was considered low (*n* = 1) and high (*n* = 2) (Table 3). At the outcome level, overall quality of the evidence was determined to be moderate for most outcomes relating to iron status and anthropometry and low or very low for remaining outcomes due to inadequate number of data points and/or high heterogeneity (Table 4).

**Table 2:** Study descriptions and main results for studies included in the review.

Reference	Population	Intervention	Control	Duration	Outcomes	Main Results
Aaron *et al.*, 2011[17]	Country: Nigeria Geography: rural Population: M and F 5–13 years (*n* = 566) Exclusion criteria: Hb < 70 g/L, signs of acute illness	RTC beverage fortified with: 11 vitamins, 12 minerals, and bioflavonoids	Iso-caloric non-fortified beverage	6 months	Hb, ferritin, retinol, zinc, anthropometry, and cognitive performance	Change in Hb, retinol and zinc significantly greater in fortified group. No significant difference between groups in Hb, ferritin, anthropometry, or cognitive performance.
Abrams *et al.*, 2003[18]	Country: Botswana Geography: urban Population: M and F 5–11 years (*n* = 311) Exclusion criteria: Hb ≤ 60 g/L, weight ≤ 15 kg, known chronic or acute illness	Beverage reconstituted from powder fortified with: 8 vitamins and 4 minerals	Iso-caloric non-fortified beverage reconstituted from powder	8 weeks	Hb, ferritin, transferrin receptors, retinol, vitamin B12, folate, riboflavin, zinc, anthropometry	Change in Hb, ferritin, folate and riboflavin; final transferrin receptors, zinc adequacy, and anthropometry significantly greater in fortified group. No significant difference between groups in retinol or vitamin B12.
Angeles-Agdeppa *et al.* 2011[10]	Country: Philippines Geography: urban Age: M and F 6-9 years (*n* = 100) Exclusion criteria: Hb > 120 g/L or Hb < 70 g/L, WAZ < −3), acute illness	RTC beverage fortified with: 2 vitamins, 2 minerals, and lysine.	Iso-caloric beverage fortified only with Vitamin C	100 days	Hb, ferritin, zinc, anthropometry, adequacy of energy and nutrient intake	Change in Hb and zinc significantly greater in fortified group. No significant difference in ferritin or anthropometry.
Ash *et al.*, 2003[19]	Country: Tanzania Geography: rural Population: M and F 6–11 years (*n* = 830) Exclusion criteria: ocular signs of xerophthalmia, Hb < 70 g/L, serious chronic disease	Beverage reconstituted from powder fortified with: 7 vitamins and 3 minerals	Iso-caloric non-fortified beverage reconstituted from powder	6 months	Hb, ferritin, erythrocyte protoporphyrin, retinol, anthropometry	Change in all measured biochemical and anthropometric indices significantly greater in fortified group.
Hyder *et al.*, 2007[15]	Country: Bangladesh Geography: rural Population: F 12 ± 1.9 years (*n* = 1125) Exclusion criteria: Hb < 70 g/L, night blindness, goiter, acute illness	Beverage reconstituted from powder fortified with: 8 vitamins and 3 minerals	Iso-caloric non-fortified beverage powder	6 months	Hb, ferritin, retinol, zinc, anthropometry	Change in Hb, ferritin, retinol, and anthropometry significantly greater in fortified group from 0–6 months, but not from 6–12 months. No significant difference between groups in zinc.
Makola *et al.*, 2003[21]	Country: Tanzania Geography: rural Population: pregnant women (*n* = 439) Exclusion criteria: Gestation <12 or > 34 weeks, Hb <80 g/L, serious medical condition or pregnancy complication	Beverage reconstituted from powder fortified with: 8 vitamins and 3 minerals	Iso-caloric non-fortified beverage powder for home reconstitution	8 weeks	Hb, ferritin, retinol, thyroid stimulating hormone	Change in Hb and ferritin significantly greater in fortified group. No significant difference between groups in retinol and thyroid stimulating hormone.
Solon *et al.*, 2003[20]	Country: Philippines Geography: urban Age: M and F grade 1–6, mean age 9.9 ± 2.2 years (*n* = 831) Exclusion criteria: Hb ≤ 80 g/L	Beverage reconstituted from powder fortified with: 8 vitamins and 3 minerals	Iso-caloric non-fortified beverage reconstituted from powder	16 weeks	Hb, urinary iodine, anthropometry, physical fitness, cognitive performance	Change in iodine significantly greater in fortified group. No significant difference between groups in Hb, anthropometry, physical fitness, or cognitive performance.
Taljaard *et al.*, 2013[14]	Country: South Africa Geography: peri-urban Age: M and F 6–11 years (*n* = 414) Exclusion criteria: health condition precluding cognitive testing, medication or supplement use	Beverage reconstituted from powder fortified with: 8 vitamins and 4 minerals (with or without nutritive sweetener; group receiving sweetened beverage included in meta-analysis)	Iso-caloric non-fortified beverage reconstituted from powder (with or without nutritive sweetener; group receiving sweetened beverage included in meta-analysis)	8.5 months	Hb, ferritin, transferrin receptors, zinc protoporphyrin, retinol, zinc, anthropometry, cognitive performance	Change in Hb, ferritin, zinc protoporphyrin, and some cognitive performance indicators significantly greater in MMN fortified groups; MMN groups w/ significantly decreased odds of iron deficiency. No significant difference between groups in transferrin receptors, retinol or zinc.
Thankachan *et al.*, 2012[11]	Country: India Geography: urban Population: M and F 6–12 years (*n* = 246) Exclusion criteria: Hb < 80 g/L, chronic illness, physical handicaps, WAZ or HAZ < −3	Beverage reconstituted from powder fortified with: 5 vitamins and 2 minerals	Iso-caloric non-fortified beverage reconstituted from powder	8 weeks	Hb, ferritin, transferrin receptors, zinc protoporphyrin, retinol, zinc, vitamin B12, RBC folate, body iron stores, vitamin C, anthropometry, morbidity	Change in Hb, ferritin, transferrin receptors, zinc protoporphyrin, retinol, vitamin B12, folate, body iron stores and vitamin C significantly greater in fortified group. No significant difference between groups in zinc, anthropometry, or morbidity.
Vaz *et al.*, 2011[13]	Country: India Geography: urban Population: M and F 7–10.5 years (*n* = 190) Exclusion criteria: Hb < 80 g/L, cardiovascular or respiratory disease, physical disability, recent history of serious infections, surgery, or injuries, nutritional supplements use	Beverage reconstituted from powder fortified with: 11 vitamins and 6 minerals	Iso-caloric non-fortified beverage reconstituted from powder, non-intervention control (placebo control only included in meta-analysis)	4 months	Ferritin, transferrin receptors, vitamin B12, vitamin C, RBC thiamin, folate, and riboflavin, pyridoxal phosphate, niacin, aerobic capacity, whole body endurance	Change in ferritin, transferrin receptors, vitamin B12, vitamin C, pyridoxal phosphate, RBC thiamin, folate, and riboflavin, aerobic capacity, and whole body endurance significantly greater in fortified group. No significant difference between groups in niacin.

**Table 3:** Methodological quality of studies included in the analysis.

Author, year	Adequate Sequence Generation?	Adequate Allocation Concealment?	Blinding	Loss to Follow-Up	Intention to Treat Analysis?	Free of Selective Reporting?	Other Bias?	Comments	Grade
Aaron, 2011 [17]	Yes, groups stratified proportionate to number of students in each school and class level	Yes, beverages identical in taste and appearance	Double blind, placebo controlled	6% (*n* = 32) due to relocation or school withdrawal	Yes	Yes	2 schools	Beverage contained maize, soy isolate and bioflavonoids in addition to MMN, de-worming 1 month prior to end point per school policy	High
Abrams, 2003[18]	No, students in 2 schools assigned to intervention and control groups, respectively	Yes, beverages identical in flavor and appearance	Double blind, placebo controlled	15% (*n* = 44), reasons for loss not stated	Yes	Yes	Lack of participant-level randomization	β-carotene rather than retinol as source of vitamin A, short (8 week) intervention period	Moderate (inadequate sequence generation)
Angeles-Agdeppa, 2011[10]	Detailed method not stated.	Not stated directly, beverages provided in foil packs	Double blind placebo controlled	11% (*n* = 11) due to relocation, school change, or absence during data collection	Not stated	Yes		Low (method of randomization not reported, all children moderately anemic at baseline)
Ash, 2003[19]	Method not stated, students stratified by median Hb prior to randomization	Yes, beverages identical in taste and appearance	Double blind, placebo controlled	7% (*n* = 56) due to poor attendance, leaving school, refusing venipuncture	Yes	Yes	Baseline ferritin differed significantly between groups	--	Moderate (method of randomization not reported)
Hyder, 2007[15]	Yes , random # assigned to participants, groups defined as even and odd	Yes, beverages identical in weight, color, flavor and appearance	Double blind, placebo controlled	12% (*n* = 136) due to missing data, illness, refusal	Yes	Yes	Parasitic infection not measured, analysis involved females only	Energy content of intervention/placebo not reported, target population adolescent girls, 0-6 month intervention period included in meta-analysis	Moderate (included F only so not generalizable)
Makola, 2003[21]	Yes, block randomization (10 subjects per block) at each of 6 study centers	Yes, beverages identical in appearance, color and taste and packaged similarly	Double blind, placebo controlled	41% (*n* = 180) due to logistic problems, early delivery	Not stated	Yes		2nd–3rd trimester pregnant women included, short (8 week) intervention period. Variable simultaneous use of iron/folic acid supplements	Moderate (high loss to follow-up)
Solon, 2003[20]	Not stated, students randomized into 4 groups (2*2, beverage*de-worming tablets)	Yes, beverages indistinguishable in appearance, smell, and taste	Double blind, placebo controlled	5% (*n* = 43), reasons not described	Not stated	Yes	Approximately half of subjects in fortified and placebo beverage group received de-worming treatment.	Effects on micronutrient status other than iron and iodine not reported. All participants included in meta-analysis by beverage group.	Moderate (method of randomization not reported)
Taljaard, 2013[14]	Not stated, randomisation to 4 groups (2*2 MMN*sugar) by school, classroom, and gender	Yes, beverages identical in color and taste.	Double blind, placebo controlled	3.9% (*n* = 16) due to leaving school	Not stated	Yes		Only iso-caloric MMN + sugar and sugar only interventions included in meta-analysis. De-worming prior to intervention	High
Thankachan, 2012[11]	Yes, block randomization with a computer-generated list in blocks of 20	Yes, beverages identical in color, size, and taste	Double blind, placebo controlled	1% (*n* = 3) due to prolonged school absence or refusal of blood draw	Not stated	Yes	Included only participants who were iron deficient at baseline (serum ferritin < 45 pmol/L)	Short (8 week) intervention period	Moderate (included only iron-deficient participants, not generalizable)
Vaz, 2011[13]	Yes, block randomization using computer generated sequence into 3 arms (MMN fortified, unfortified, and no beverage)	Not stated, MMN fortified and unfortified beverages both choco-malt	Double blind placebo controlled	4% (*n* = 13) due to withdrawal of consent	Yes	Yes	Baseline ferritin differed significantly between MMN fortified and unfortified beverage groups	Only iso-caloric MMN fortified and unfortified interventions included in meta-analysis	Moderate (allocation concealment not reported directly)

**Table 4:** Summary of findings and overall assessment of quality of evidence grade by study outcome.

Quality Assessment						Summary of Findings		
No. Studies	I^2^ (%)	Heterogeneity	Generalizable to Population of Interest?	Generalizable to Intervention of Interest?	Other Sources of Bias (e.g., Major Limitations in Study Design)	No. Participants	Publication Bias r (*p*-value)	Effect Estimate
Hemoglobin (g/L): Overall quality of evidence grade = moderate								
8	92	6 of 8 studies found significantly greater increase in Hb in intervention group. Other studies found no difference between groups.	7 of 8 studies conducted in M and F school age children in lower-income countries. 1 study in F adolescents in Bangladesh.	Variability in micronutrient composition, dose, and duration. Reconstituted powder used in 6 of 8 studies.	Study w/highest iron dose found no difference between groups, several randomized by school, de-worming protocols inconsistent.	3835	0.25 (0.56)	2.76 [1.19, 4.33]
Ferritin (pmol/L): Overall quality of evidence grade = moderate								
8	95	6 of 8 studies found significantly greater increase in ferritin in intervention group. Other studies found no significant difference between groups.	7 of 8 studies conducted in M and F school age children in lower-income countries. 1 study in F adolescents in Bangladesh.	Variability in micronutrient composition, dose, and duration. Reconstituted powder used in 6 of 8 studies.	Study w/highest iron dose found no difference between groups, baseline ferritin differed in 1 study, several randomized by school, de-worming protocols inconsistent.	3891	0.94 (0.004)	15.42 [5.73, 25.12]
Retinol (µmol/L): Overall quality of evidence grade = low								
5	61	3 of 5 studies found significantly greater increase in retinol in intervention group. Other studies found no significant difference between groups.	4 of 5 studies conducted in M and F school age children in lower-income countries. 1 study in F adolescents in Bangladesh.	Variability in micronutrient composition, dose, and duration. Reconstituted powder used in 4 of 5 studies.	Randomization method unclear or at school level in some studies, de-worming protocols inconsistent.	2049	0.30 (0.62)	0.05 [−0.03, 0.13]
Zinc (µmol/L): Overall quality of evidence grade = low									
4	75	2 of 4 studies found significantly greater increase in zinc in intervention group. Other studies found no significant difference between groups.	3 of 4 studies conducted in M and F school age children in lower-income countries. 1 study in F adolescents in Bangladesh.	Variability in micronutrient composition, dose, and duration. Reconstituted powder used in 2 of 4 studies.	Randomization method not explicit in 2 studies, de-worming protocols inconsistent.	1690	0.09 (0.91)	0.92 [−1.45, 3.30]	
Vitami n B12 (pmol/L): Overall quality of evidence grade = very low									
3	99	2 of 3 studies found significantly greater increase in vitamin B12 in intervention group. Other study found no differences between groups.	All studies conducted in M and F school children in lower-income countries.	Variability in micronutrient composition, dose, and duration. Reconstituted powder used in all studies	One study randomized by school, infection and/or parasites not treated or measured consistently.	644	0.91 (0.27)	96.2 [−142.2, 334.6]	
Weight (kg): Overall quality of evidence grade = moderate									
6	85	3 of 6 studies found significantly greater increase in vitamin B12 in intervention group. Other studies found no differences between groups.	5 of 6 studies conducted in M and F school age children in lower-income countries. 1 study in F adolescents in Bangladesh.	Variability in micronutrient composition, dose, and duration. Reconstituted powder used in all studies.	Randomization method unclear or at school level in 4 studies, de-worming protocols inconsistent.	2977	0.04 (0.94)	0.30 [0.01,0.58]	
Height (cm): Overall quality of evidence grade = moderate								
5	78	2 of 5 studies found significantly greater increase in vitamin B12 in intervention group. Other studies found no differences between groups.	4 of 5 studies conducted in M and F school age children in lower-income countries. 1 study in F adolescents in Bangladesh.	Variability in micronutrient composition, dose, and duration. Reconstituted powder used in 4 of 5 studies.	Randomization not outlined specifically in 3 studies, de-worming protocols inconsistent.	2697	0.12 (0.85)	0.17 [−0.16, 0.50]
Weight-for-age (Z): Overall quality of evidence grade = low								
4	59	2 of 4 studies found significantly greater increase in vitamin B12 in intervention group. Other studies found no differences between groups.	All studies conducted in M and F school children in lower-income countries.	Variability in micronutrient composition, dose, and duration. Reconstituted powder used in 3 of 4 studies.	Method of randomization not specific or at school level in all studies, de-worming protocols inconsistent.	1385	0.65 (0.35)	0.028 [−0.06, 0.12]
Height-for-age (Z): Overall quality of evidence grade=moderate								
3	0	No significant differences found between groups	All studies conducted in M and F school children in lower-income countries.	Variability in micronutrient composition, dose, and duration. Reconstituted powder used in 2 of 3 studies.	Method of randomization not specific or at school level in all studies, de-worming protocols inconsistent.	1124	0.10 (0.94)	0.0 [−0.05, 0.05]
Anemia (Hb < 110–120 g/L): Overall quality of evidence grade = moderate									
6	84	5 of 6 studies found significantly greater reduction in endpoint prevalence of anemia in intervention *vs.* control group.	5 of 6 studies conducted in M and F children in lower-income countries. 1 study in F adolescents in Bangladesh.	Variability in micronutrient composition, dose, and duration. Reconstituted powder used in 4 of 6 studies.	Method of randomization not specific or at school level in 3 studies, de-worming protocols inconsistent. Study w/highest iron dose only to find no significant difference between groups in anemia reduction.	2828	0.24 (0.64)	RR 0.63 [0.54, 0.73]	
Iron deficiency (ferritin < 27–45 pmol/L): Overall quality of evidence grade = moderate									
7	96	4 of 7 studies found significantly greater reduction in endpoint prevalence of iron deficiency in intervention *vs.* control group	6 of 7 studies conducted in M and F children in lower-income countries. 1 study in F adolescents in Bangladesh.	Variability in micronutrient composition, dose, and duration. Reconstituted powder used in 5 of 7 studies.	Method of randomization not specific or at school level in 3 studies, de-worming protocols inconsistent.	2523	0.23 (0.62)	RR 0.32 [0.23,0.45]	
Iron deficiency anemia (Hb < 110–120 g/L and ferritin < 27–45 pmol/L): Overall quality of evidence grade = low									
3	97	2 of 3 studies found significantly greater reduction in endpoint prevalence of IDA in intervention *vs.* control group.	2 of 3 studies conducted in M and F children in lower-income countries. 1 study in F adolescents in Bangladesh.	Variability in micronutrient composition, dose, and duration. Reconstituted powder used in2 of 3 studies.	De-worming protocols inconsistent.	1649	0.88 (0.31)	RR 0.13 [0.07,0.25]	

### 3.3. Meta-Analysis

Results of all trials conducted in children (*n* = 9, total sample size = 4094) were included in the meta-analyses. Compared with controls who received a non-fortified beverage, children who received MMN-fortified beverages for a duration of 8 weeks to 6 months demonstrated significantly greater improvements in circulating hemoglobin (+2.76 g/L, 95%-CI [1.19, 4.33], *p* = 0.004; 8 studies) and ferritin (+15.42 pmol/L, [5.73, 25.12], *p* = 0.007; 8 studies). Exclusion of an anomalous study that found no change in hemoglobin or ferritin in either intervention or control groups did not significantly alter these results [17]. No significant intervention effects of fortified beverages were found for serum retinol (+0.05 μmol/L [−0.03, 0.13], *p* = 0.16; 5 studies), zinc (+0.92 µmol/L [−1.45, 3.30], *p* = 0.30; 4 studies), or vitamin B12 (+96.2 pmol/L [−142.2, 334.6], *p* = 0.22; 3 studies). 

A moderate but significant effect was found for weight gain in the intervention compared with the control groups (+0.30 kg [0.01,0.58], *p* = 0.04; 6 studies), while neither height gain nor weight-for-age or height-for-age *Z*-scores differed significantly between groups (+0.17 cm [−0.16, 0.50], *p* = 0.23, 5 studies; +0.028 [−0.06, 0.12], *p* = 0.40, 4 studies; and 0.0 [−0.05, 0.05], *p* = 0.98, 3 studies respectively). Although the number of data points precluded meta-analysis, we found some evidence of a favorable effect of supplementation with MMN-fortified beverages on riboflavin status [13,18], vitamin B6 [13], vitamin C [11,13], thiamin [13], folate [11,13], urinary iodine [7], body mass index [4,15], and mid-upper arm circumference [4,15]. For indices of physical performance results were inconsistent; one trial found significant improvement in aerobic capacity and endurance in intervention compared with control groups [13], while no difference in fitness index score or push up capacity between groups was found in a separate trial [7]. Two trials found no effects on cognitive indices [7,17], while one found gains in some cognitive test scores in the MMN-fortified group [14]. No effects were found on circulating niacin [13] or indices of morbidity [11].

**Figure 1:**
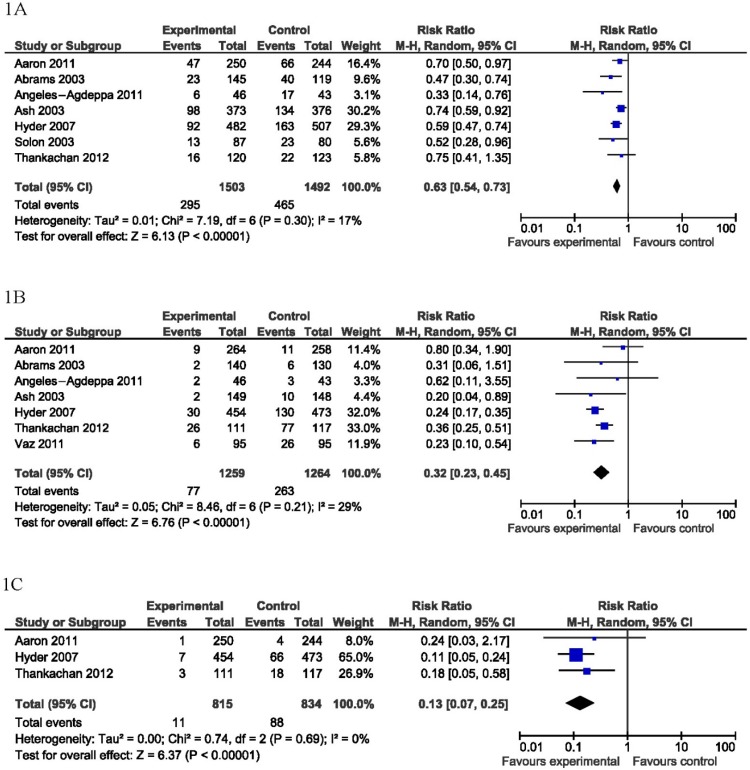
Forest plot of studies assessing hemoglobin and iron outcomes. Consumption of MMN beverages fortified compared to iso-caloric controls significantly reduced the risk of anemia (**1A**); iron deficiency (**1B**); and iron deficiency anemia (**1C**).

Consumption of fortified beverages significantly reduced the risk of anemia (RR 0.63 [0.54, 0.73], *p* < 0.001; *n* = 6 studies, defined as Hb < 110–120 g/L), iron deficiency (RR 0.32 [0.23,0.45], *p* < 0.001; *n* = 7 studies, defined as ferritin <27−45 pmol/L), and iron deficiency anemia (RR 0.13 [0.07, 0.25], *p* < 0.001; *n* = 3 studies, defined as a combination of biochemical criteria for iron deficiency and anemia) (Figure 1). Fortified beverage consumption did not significantly alter the risk of vitamin A or zinc deficiency.

Meta-regression analyses were conducted for outcomes with at least five data points from different trials (ferritin, hemoglobin and weight). Considered independently, daily iron dose, total iron dose (daily dose x duration), intervention duration, baseline status, and deworming (binary Y/N) were not significantly associated with the difference in ferritin change between groups. A significant inverse association was found between daily iron dose and hemoglobin change (β = −0.4, *p* = 0.02) with all studies included in analysis. The association was attenuated when total iron dose was substituted for daily iron dose (β = −0.001, *p* = 0.06). However, the significance of this relationship did not persist after excluding the study by Aaron *et al.* (*p* > 0.90) [17]. Baseline hemoglobin, intervention duration, and deworming were not predictive of change in hemoglobin, nor were baseline weight, intervention duration, or deworming predictive of differences in weight change. No adjustments were made for compliance rate, which was reported in only four studies.

Study duration was not significantly associated with any of the biochemical or anthropometric outcomes measured. When comparing reconstituted powder to RTC beverages, there were no significant differences in any index except for ferritin, which demonstrated significantly less change in interventions with RTC beverages. However, after excluding the study by Aaron *et al.*, differences were no longer significant.

A single placebo-controlled trial evaluated an 8-week intervention with a non-dairy MMN-fortified beverage in Tanzanian women who were 12–34 weeks gestation at baseline [21]. Only women who had not delivered by the end of the study period were included in analysis. Compared with women in the control group, there was a significant improvement in hemoglobin (*p* = 0.015) and serum ferritin (*p* = 0.009) but not in serum retinol in women in the intervention group. Consumption of iron/folic acid tablets did not differ significantly between the two groups. 

## 4. Discussion

To the best of our knowledge, this is the first systematic review and meta-analysis to focus on nutritional outcomes related to consumption of MMN fortified beverages in the context of low-middle income countries. Among school children who consumed non-dairy MMN fortified beverages compared with controls consuming iso-caloric non-fortified placebo beverages, results from the meta-analysis showed significant improvements in hemoglobin and ferritin, but not in other biochemical indices. Consequently, consumption of fortified beverages significantly reduced the risk of anemia, iron deficiency, and iron deficiency anemia, but did not significantly alter the risk of other micronutrient deficiencies. In the meta-regression analyses, baseline status, intervention dose, study duration, and deworming were not significant predictors of differences in hemoglobin, ferritin, or weight change between groups. 

A small but significant effect was found for weight gain in micronutrient *vs.* control groups despite beverages in both groups being iso-caloric. No effects were found on other anthropometric indices. Morbidity and functional outcomes, such as cognition and physical performance, could not be adequately assessed due to insufficient data points. Although meta-analysis was precluded by the scarcity of data, findings from a single MMN-fortified beverage trial conducted in pregnant women in Tanzania were consistent with results of the meta-analysis in school children.

Results from the present analysis are in general agreement with the findings from a previous systematic review on MMN fortification of foods or beverages in school children [22]. Eleven of the 12 studies in the previous systematic review were conducted in transitional or developing countries, and four of these overlapped with studies included in the present meta-analysis [4,7,14,15]. Although meta-analyses were not performed, the investigators reported an improvement in hemoglobin and iron status in eight of the 10 studies in which micronutrient outcomes were measured, as well as a reduction in anemia in seven studies. Beneficial effects on cognitive performance were found in two studies of longer duration (6 and 12 months).

Findings from the present study were similar to a recently published Cochrane review on the effects of micronutrient powders (MNP) among infants and children (6–23 months at the time of the intervention) in low-income countries [23]. In the Cochrane analysis, home fortification with MNP was shown to reduce the likelihood of anemia by 31% and iron deficiency by 51% compared with no intervention or placebo, but did not have an effect on growth measured by weight-for-age, length-for-age, and weight-for-length Z-scores. A single study included in the review reporting serum zinc as an outcome did not find an effect of MNP including 5 mg/d zinc for 6 months on zinc concentration [24]; retinol and vitamin B12 were not reported as outcomes.

The greater impact of MMN-fortified beverages on anemia and iron status compared with other biochemical outcomes in this as well as previous reviews [22,23] may have several explanations. Inconsistency in biochemical markers of micronutrient status precluded meta-analysis for several micronutrients that did show positive impacts in individual studies included in the current review. Among outcomes for which a meta-analysis was feasible, hemoglobin and ferritin were the most common nutritional indices measured, yielding more data points and higher power in the analysis. Furthermore, methodological limitations, including the validity of serum retinol as an index of status [25], poor sensitivity of plasma zinc [8], and differences in laboratory techniques used to measure circulating vitamin B12, may have obscured positive findings for other micronutrients.

Despite the stronger grade of evidence for hemoglobin and iron status outcomes in the present analysis, evidence suggests that combined MMN supplementation may have a greater effect than iron supplementation alone. A meta-analysis on the effects of micronutrients on growth in children below age 18 years found that iron alone had no significant effect but that MMN interventions improved linear and possibly ponderal growth [26]. This phenomenon is likely related to the frequent presence of MMN deficiencies in children in developing countries subjected to poor dietary quality coupled with frequent episodes of infection. 

Several considerations should be made with regard to implementing beverage fortification strategies. Firstly, as with any nutrition strategy, the justification for fortifying a vehicle should be based on demonstrated micronutrient needs and health parameters of the population, and should take into account issues such as safe administration of micronutrient interventions [6]. In higher-income countries fortification has come under criticism due to some products on the market being fortified with micronutrients that are not necessarily deficient in the diet [27]. This practice is misleading to consumers, and may result in excessive consumption of a product due to perceptions of its health benefits. Second, there is a growing body of literature on the negative effects of sugar-sweetened beverages. While it is beyond the scope of the current research to cover this topic, considerations to limit the caloric content of beverages selected for fortification are critical due to the increasing double burdens of under- and over-nutrition in many low-middle income countries [28]. In the present meta-analysis, weight gain was modestly but significantly greater in the groups receiving MMN fortified beverages compared with controls despite the fact that the beverages were iso-caloric. This finding may suggest that specific micronutrients or their combination could have contributed to the modest weight gain observed. Most studies did not report the energy content of beverages provided, which precluded this factor being assessed in the meta-analysis. However, a single study in South Africa comparing caloric with non-caloric MMN-fortified beverages found an inverse association between sugar as a nutritive sweetener and weight-for-age *Z*-score [14].

Strengths of the present systematic review and meta-analysis include a comprehensive and targeted search strategy to identify relevant publications and a thorough quantitative analysis of available data. Limitations were the small number of beverage fortification RCT, multiple reported outcomes with insufficient data points for inclusion in meta-analysis, and incomplete information on quantities and chemical forms of micronutrients, quantities of macronutrients (particularly energy and protein), and compliance. However, despite the aforementioned limitations, results from the analyses showed a clear benefit of MMN-fortified beverage fortification on anemia and iron status, both of which are high burden global public health issues. 

## 5. Conclusions

In summary, evidence from the present study suggests that the administration of non-dairy MMN-fortified beverages in the context of school settings in low-middle income countries is effective at improving hemoglobin and ferritin, and reducing the prevalence of anemia, iron deficiency, and iron deficiency anemia. While only one study evaluated the impacts on women, results were consistent with the broader findings in meta-analyses among children. Despite the positive impacts found in the present review, considerations to limit the caloric content of beverages selected for fortification are important if used in programs or market-based settings. Additional research is needed to investigate the effects of MMN-fortified beverages on other biochemical outcomes and population subgroups, as well as to evaluate effectiveness in programmatic settings.

## Supplementary Files

Supplemental Figure S1—Consort flow diagram of study article selection and inclusion process. Supplemental Table S1—Micronutrient formulations for studies included in the systematic review and meta-analysis.
