# Supplementary material for: Multiple-Micronutrient Fortified Non-Dairy Beverage Interventions Reduce the Risk of Anemia and Iron Deficiency in School-Aged Children in Low-Middle Income Countries: A Systematic Review and Meta-Analysis (i–iv)
Source: Nutrients. 2015 May 21;7(5):3847–68. doi: 10.3390/nu7053847 (PMC4446783; doi:10.3390/nu7053847)
Supplement: Supplementary file 1 [file nutrients-07-03847-s001.docx]

**Supplementary Information**

**Table S1.** Micronutrient formulations for studies included in the systematic review and meta-analysis.

| **Nutrient** | **Aaron, 2011** | **Angeles-Agdeppa, 2011** | **Vaz, 2011** | **Hyder, 2007** | **Abrams, 2003** | **Ash, 2003** | **Makola, 2003** | **Solon, 2003** | **Thankachan, 2012** | **Taljaard, 2013** |
| --- | --- | --- | --- | --- | --- | --- | --- | --- | --- | --- |
| Energy (kcal) | 164.6 | 106 |  |  |  |  | 87.9 |  |  | 81 |
| Carbohydrates (g) | 26.4 |  |  |  |  |  |  |  |  | 20.6 |
| Dietary fiber (g) | 2.5 |  |  |  |  |  |  |  |  |  |
| Fat (g) | 4.1 |  |  |  |  |  |  |  |  |  |
| Protein (g) | 5.2 |  |  |  |  |  |  |  |  |  |
| Vitamin A (µg RE) | 1000 | 133.3 | 250 | 389 | 400 | 525 | 1050 | 210 | 240 | 400 |
| Vitamin B12 (Cobalamin) (µg) |  |  | 2.3 | 1 | 1 | 3 | 6 | 0.5 | 1.27 | 1 |
| Biotin (µg) | 47 |  | 10 |  |  |  |  |  |  |  |
| Vitamin C (mg) | 60 | 45 | 75.2 | 120 | 60 | 72 | 144 | 75 | 27 | 60 |
| Vitamin D (µg) | 1.2 |  | 1.3 |  |  |  |  |  |  |  |
| Vitamin E (mg) | 6.5 |  |  | 10 | 7.5 | 10.5 | 21 | 2.5 |  | 7.5 |
| Folate (µg) | 200 |  | 225 | 120 | 140 | 140 | 280 | 60 | 35 | 140 |
| Niacin (B3) (mg) | 18 |  | 12 | 5 | 2.7 | 5 | 10 | 2.5 |  | 2.7 |
| Pantothenic acid (B5) (mg) | 2.3 |  | 2 |  |  |  |  |  |  |  |
| Pyridoxine (B6) (mg) | 2 |  | 1.3 | 1 | 0.5 | 0.7 | 1.4 | 0.5 |  | 0.5 |
| Riboflavin (B2) (mg) | 1.6 |  | 1.1 | 0.91 | 0.4 | 0.6 | 1.2 | 0.46 | 0.63 | 0.4 |
| Thiamine (B1) (mg) | 1 |  | 1.1 |  |  |  |  |  |  |  |
| Calcium (mg) | 84 |  | 231 |  | 120 |  |  |  |  | 120 |
| Copper (mg) | 1.2 |  | 350 |  |  |  |  |  |  |  |
| Iodine (µg) | 150 |  | 79.2 | 75 | 60 | 45 | 90 | 48 |  | 60 |
| Iron (mg) | 14 | 1.3 | 17.8 | 7 | 7 | 5.4 | 10.8 | 4.8 | 5.9 | 7 |
| Magnesium (mg) | 49 |  | 33 |  |  |  |  |  |  |  |
| Manganese (mg) | 4.5 |  |  |  |  |  |  |  |  |  |
| Molybdenum (µg) | 29 |  |  |  |  |  |  |  |  |  |
| Phosphorus (mg) | 187 |  |  |  |  |  |  |  |  |  |
| Potassium (mg) | 276 |  |  |  |  |  |  |  |  |  |

**Table S1.** *Cont.*

| **Nutrient** | **Aaron, 2011** | **Angeles-Agdeppa, 2011** | **Vaz, 2011** | **Hyder, 2007** | **Abrams, 2003** | **Ash, 2003** | **Makola, 2003** | **Solon, 2003** | **Thankachan, 2012** | **Taljaard, 2013** |
| --- | --- | --- | --- | --- | --- | --- | --- | --- | --- | --- |
| Selenium (µg) | 24.8 |  |  |  |  |  |  |  |  |  |
| Vanadium (µg) | 25 |  |  |  |  |  |  |  |  |  |
| Zinc (mg) | 15 | 1.4 | 1.8 | 7.5 | 3.75 | 5.25 | 10.5 | 3.75 | 1.2 | 3.75 |
| Bioflavonoids (mg) | 87.5 |  |  |  |  |  |  |  |  |  |
| Lysine (mg) |  | 200 |  |  |  |  |  |  |  |  |

**Figure S1.** Consort flow diagram of study article selection and inclusion process.
